# Targeting nuclear receptors in muscular dystrophies and regenerative myogenesis

**DOI:** 10.3389/fcell.2026.1878702

**Published:** 2026-06-15

**Authors:** Eira Mann, Hoang Vu, Vihang A. Narkar, Yu Liu

**Affiliations:** 1 Department of Biology and Biochemistry, College of Natural Sciences and Mathematics, University of Houston, Houston, TX, United States; 2 Brown Foundation Institute of Molecular Medicine, McGovern Medical School, UTHealth, Houston, TX, United States; 3 Institute of Muscle Biology and Cachexia, University of Houston, Houston, TX, United States

**Keywords:** muscular dystrophy, myopathy, nuclear receptors, signaling, transcription factors

## Abstract

Skeletal muscle is a highly plastic tissue with a robust capacity for regeneration, largely driven by resident satellite cells. Muscular dystrophies comprise a heterogeneous group of inherited disorders characterized by progressive muscle degeneration, chronic inflammation, and impaired regenerative capacity. Despite well-defined genetic etiologies, effective disease-modifying therapies for these disorders, as well as many acquired myopathies, remain limited. Emerging evidence identifies nuclear receptors (NRs) as key regulators of skeletal muscle homeostasis, integrating hormonal, metabolic, and environmental signals to control transcriptional programs governing mitochondrial function, metabolism, inflammation, and myogenesis. In this review, we summarize the diverse roles and mechanisms of action of NRs in skeletal muscle biology and discuss how their dysregulation contributes to muscle wasting and disease progression. We also highlight emerging NR-targeted therapeutic strategies aimed at enhancing metabolic function, suppressing inflammation and fibrosis, and promoting muscle regeneration. Finally, we outline critical knowledge gaps and future directions to advance the translation of NR-based therapies for muscular dystrophies and related neuromuscular disorders.

## 1 Introduction

Skeletal muscle is a highly dynamic tissue that adapts its mass, metabolism, and contractile properties in response to exercise and environmental cues. It plays essential roles in posture, locomotion, respiration, and whole-body metabolism, while also serving as a reservoir of amino acids during energy stress. In addition, skeletal muscle functions as an endocrine organ by secreting myokines that regulate systemic physiology (Hoffmann and Weigert, 2017).

Muscle mass is maintained through a balance between protein synthesis and degradation, regulated by factors such as exercise, nutrition, hormonal signaling, aging, and disease (Joshi et al., 2025). Skeletal muscle also exhibits a remarkable capacity for regeneration driven by satellite cells, which activate, proliferate, and differentiate to repair damaged fibers while self-renewing to maintain the stem cell pool (Forcina et al., 2019). Disruption of these processes leads to muscle wasting, a hallmark of many inherited and acquired myopathies, and a major contributor to disability and mortality (Joshi et al., 2025; Allenbach and Benveniste, 2026; Zhong et al., 2024).

Muscular dystrophies are a heterogeneous group of genetic disorders characterized by progressive muscle degeneration. They arise from mutations in genes encoding structural, signaling, and nuclear proteins, with the most severe forms linked to defects in the dystrophin-glycoprotein complex (DGC) (Blake et al., 2002; Emery, 1998; Hoffman et al., 1987). Notably, the most severe forms of muscular dystrophy arise from mutations affecting components of the dystrophin-glycoprotein complex (DGC), a molecular scaffold localized to the sarcolemma that provides mechanical stability to striated muscle. For instance, Duchenne muscular dystrophy (DMD), which afflicts ∼1 out 5,000 male birth is a severe form of muscular dystrophy that results from partial or complete loss of functional dystrophin. Dystrophin is an essential component of the DGC, linking the cytoskeleton of muscle fibers to the ECM. In the absence of dystrophin, the DGC becomes functionally compromised, and the mechanical stress generated during muscle contraction leads to sarcolemma damage and myofiber necrosis (Shin et al., 2013).

Despite advances in gene therapy, including AAV-mediated micro-dystrophin delivery, significant technical and safety challenges remain (Happi Mbakam et al., 2022; Choi and Koo, 2021; Ryan, 2022; Elangkovan and Dickson, 2021; Hoffman, 2020; Novak et al., 2017; Mullard, 2023; D'Ambrosio and Mendell, 2023; Lek et al., 2023; Qiu et al., 2023). Accordingly, effective treatment of DMD and related disorders will likely require complementary approaches that reduce muscle damage and inflammation while enhancing regeneration (Robert et al., 2023). In this context, nuclear receptors (NRs) have emerged as promising regulators of muscle metabolism, vascularization, and repair. This review summarizes the roles of NRs in skeletal muscle disorders, especially muscle dystrophy and highlights emerging NR-targeted therapeutic strategies. While DMD represents the most extensively studied model, this review also incorporates available evidence from other muscular dystrophies, including limb-girdle muscular dystrophies, facioscapulohumeral muscular dystrophy, myotonic dystrophy, and dysferlinopathy. However, it is notable that mechanistic and therapeutic insights into NR signaling remain disproportionately derived from DMD-focused studies, reflecting current gaps in the broader field.

## 2 Overview of nuclear receptors

NRs are a superfamily of transcription factors that are activated by physiological ligands, such as steroid hormones, lipids, vitamins, and metabolic intermediates, as well as by xenobiotic compounds. The family also comprises orphan nuclear receptors for which endogenous ligands have not yet been identified. NRs control diverse biological functions, including inflammation, immune responses, cell fate determination, and metabolism (Frigo et al., 2021; Jin et al., 2025). NRs share several conserved structural domains, including an N-terminal activation function 1 (AF-1) domain, a central DNA-binding domain (DBD), a hinge region, and a C-terminal ligand-binding domain (LBD) that contains activation function 2 (AF-2). The AF-1 and AF-2 domains mediate interactions with various nuclear cofactors, such as coactivators and corepressors, CREB-binding protein/p300, and histone deacetylase/acetylases, which are essential for NR transcriptional activity in both ligand-dependent and ligand-independent contexts. The DBD typically consists of two zinc finger motifs that recognize and bind specific DNA response elements present within the promoters or enhancers of target genes. The response element of NRs contains core 5′-AGGTCA-3′ half-site sequences that are arranged as direct repeats, inverse repeats, or evert repeats (Forman and Evans, 1995). The LBD is unique to each NR which is responsible not only for ligand recognition but also for post-translational modifications that facilitate interactions with co-regulatory proteins, thereby modulating specific transcriptional outcomes (Becares et al., 2017; Helzer et al., 2015; Malbeteau et al., 2022).

**FIGURE 1:**
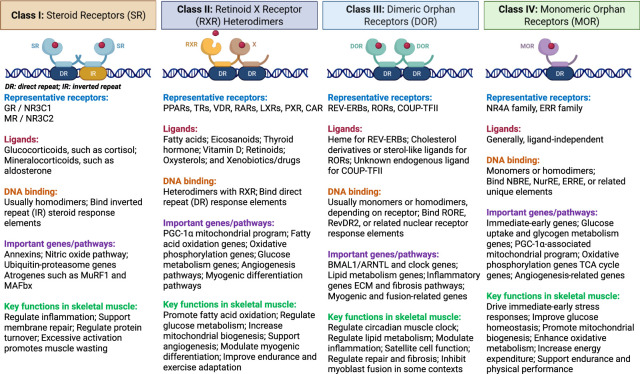
Classification and mode of action of NRs in skeletal muscle. Major classes of NRs, ligand, mechanisms and key functions in skeletal muscle are depicted here. GR, glucocorticoid receptor; MR, Mineralocorticoid receptor; PPAR, Peroxisome proliferator-activated receptor; TRs, Thyroid hormone receptors; BMAL1, Brain and Muscle ARNT-Like 1; COUP-TFII, Chicken ovalbumin upstream promoter-transcription factor II; ERR, Estrogen-related receptor; PGC-1α, Peroxisome proliferator-activated receptor gamma coactivator 1-alpha; RAR, Retinoic acid receptor; RORα, Retinoic acid receptor-related orphan receptor alpha; RXR, Retinoid X receptor; TR, Thyroid hormone receptor; VDR, Vitamin D receptor; LXRs, Liver X receptors; PXR, Pregnane X receptor; CAR, Constitutive androstane receptor. The image was created with BioRender.com.

The NR superfamily comprises 48 members which are classified into numbered subfamilies (NR1-NR6, plus orphan NR0) based on sequence homology and evolutionary relationships (Frigo et al., 2021). Functionally, NRs are subdivided into four major groups attributed to their ligand-binding properties, DNA-binding specificities, and dimerization patterns (Mangelsdorf and Evans, 1995; Mangelsdorf et al., 1995) (Figure 1). Class I receptors are hormone-activated steroid receptors which are localized in cytoplasm and move to nucleus after binding with their ligands. They function as homodimers and bind to inverted repeat half-site sequences located within the promoters or enhancers of target genes. Classical steroid receptors, including estrogen, progesterone, androgen (testosterone), glucocorticoid, and mineralocorticoid receptors, belong to this subgroup. Class II receptors typically form obligate heterodimers with retinoid X receptors (RXRs) and are activated by non-steroidal hormones. Some of the examples of class II receptors include, peroxisome proliferator-activated receptors (PPARs), thyroid hormone receptors (TRs), vitamin D receptor (VDR), retinoic acid receptors (RARs), liver X receptors (LXRs), pregnane X receptor (PXR), and constitutive androstane receptor (CAR). This NR subgroup recognizes direct repeat half-site sequences and are predominantly localized in the nucleus, even in the absence of ligand. Class III receptors, function as homodimers and bind to direct repeat half-site sequences. While originally classified as orphan receptors, some of them have since been shown to bind endogenous or metabolic ligands, such as fatty acids. Finally, class IV receptors are orphan receptors that bind unique DNA sequences, such as nerve growth factor-inducible element binding (NBRE) sites, either as monomers or as components of complex dimers (Frigo et al., 2021; Jin et al., 2025).

## 3 Nuclear receptor signaling in muscle diseases

Accumulating evidence indicates that NRs regulate metabolic homeostasis and influence disease progression through mechanisms extending beyond classical transcriptional control, such as epigenetic modifications and interactions with other proteins (Jin et al., 2025). Impaired NR signaling is linked to a variety of disorders, including cancer, type 2 diabetes, cardiovascular disorders, and many conditions involving inflammation and tissue breakdown, highlighting their importance as potential therapeutic targets. Indeed, due to their conserved ligand-binding domains, which are amenable to pharmacological modulation, NRs remain attractive targets across diverse therapeutic areas (Huang and Glass, 2010; Montaigne et al., 2021; Paredes et al., 2021). Available literature also suggests that NRs are major regulators of skeletal muscle oxidative phosphorylation and fatty acid oxidation, mitochondrial content, angiogenesis, and exercise tolerance (Kumar and Narkar, 2023). Indeed, several NR-targeting agents have advanced into clinical evaluation, particularly those modulating glucocorticoid and mineralocorticoid receptor pathways (Table 1). However, the roles and full therapeutic potential of modulating various NRs in inherited and acquired muscle disorders remain incompletely understood. In the following sections, we discuss the mechanisms of action of various NRs and their potential to be used as molecular targets for treatment of various muscular diseases.

**TABLE 1:** Status of clinical trials targeting nuclear receptors in muscular dystrophies.

Target NR	Drug	Clinical trial (official title/Identifier)	Status/Phase	Diseases	Status/Outcome
GR	Prednisone/prednisolone	In clinical use	Standard of care	DMD	Improves skeletal muscle strength; prolongs ambulation; long-term use causes major side effects
GR	Vamorolone	VISION-DMD: A study to assess the efficacy and safety of vamorolone (NCT03439670)	Phase IIb	DMD	Completed; efficacy comparable to prednisone with improved safety
GR	Vamorolone	VBP15-006: Safety and PK study in young boys with DMD	Phase II	DMD	Completed; well tolerated with functional improvement signals
GR	Vamorolone	Long-term extension study	Open-label extension	DMD	Sustained benefits with reduced steroid-associated adverse effects
MR	Eplerenone	E-SCAR DMD: Eplerenone for subclinical cardiomyopathy in DMD (NCT01521546)	Phase II (RCT)	DMD cardiomyopathy	Completed; preserved cardiac function
MR	Eplerenone ± spironolactone	Therapeutic potential for aldosterone inhibition in DMD (NCT02354352)	Phase III	DMD	Completed; supports cardioprotective effects
GR (steroid class)	Deflazacort	Phase 3B study of deflazacort in DMD (PTCEMF) (NCT03642145)	Phase IIIb	DMD	Withdrawn; efficacy already established
GR (steroid class)	Deflazacort	FOR-DMD: Finding the optimum regimen for DMD (NCT01603407)	Phase III	DMD	Completed; informed corticosteroid dosing strategies
PPARδ	ASP0367 (bocidelpar)	MOUNTAINSIDE trial (NCT04641962)	Phase II	Mitochondrial myopathy	Terminated; failed efficacy despite acceptable safety
PPARδ	ASP0367	Adaptive phase II/III MOUNTAINSIDE program	Phase II/III	Mitochondrial myopathy	Development discontinued due to lack of clinical benefit
VDR	Vitamin D/analogues	Observational and interventional studies (not NR-targeted trials)	Early/adjunct	DMD and other myopathies	Evaluated mainly as supportive therapy; No dedicated NR-targeted clinical trials

### 3.1 Glucocorticoid receptor (GR)

The GR is an evolutionarily conserved member of the Class I NRs which functions as a ligand-dependent transcription factor (Figure 1). Endogenous glucocorticoids, such as cortisol (hydrocortisone), as well as synthetic steroids, including prednisone and dexamethasone, bind and activate GR, allowing it to translocate to nucleus where it binds to consensus glucocorticoid response element (GRE) DNA sequence present in the promoter regions of various genes (Luisi et al., 1991). GR bound to glucocorticoid can also reduce transcription through association with negative GREs, which allows recruitment of corepressors and histone deacetylases (Surjit et al., 2011). Glucocorticoids are critical for maintaining physiological homeostasis which is also evidenced by the findings that most of the global GR knockout mice die at birth or shortly after due to respiratory insufficiency (Whirledge and DeFranco, 2018). Moreover, tissue-specific GR knockout mice have provided strong evidence that GR plays critical roles in lung development, metabolism, inflammation, and neurological health (Whirledge and DeFranco, 2018; Macfarlane et al., 2024). Glucocorticoids are now widely used therapeutically in the management of autoimmune, inflammatory, allergic, and lymphoproliferative diseases (Whirledge and DeFranco, 2018).

Pharmacological glucocorticoids, including prednisone and deflazacort, are the standard of care for patients with DMD (Table 1). Numerous independent studies have demonstrated that glucocorticoid therapy prolongs ambulation, reduces the incidence of scoliosis, and improves cardiopulmonary function, quality of life, and overall survival (McDonald et al., 2018; Hussein et al., 2010; Bach et al., 2010). At the cellular level, glucocorticoids reduce muscle fiber degeneration and necrosis as well as dampens chronic inflammatory responses in dystrophic muscle (Quattrocelli et al., 2021). One of the mechanisms by which glucocorticoids provide functional improvement in dystrophic muscle through direct induction of the metabolic transcription factor Kruppel-like factor 15 which enhances nutrient utilization (Morrison-Nozik et al., 2015). Glucocorticoid steroids also promote sarcolemmal repair through directly increasing the gene expression of annexins A1 and A6, which mediate myofiber repair. (Quattrocelli et al., 2017). In addition, glucocorticoids stimulate the L-arginine/nitric oxide pathway, leading to increased nitric oxide production, improved endothelial function, and attenuation of muscle ischemia and hypoxia in DMD (Horster et al., 2015). Moreover, glucocorticoids can also increase the expression of utrophin, a homolog of dystrophin which improves sarcolemma stability in dystrophic muscle (Miura et al., 2008).

Glucocorticoids exert potent anti-inflammatory effects by repressing nuclear factor-kappa B and activator protein-1 transcription factors, thereby reducing cytokine production (Vandev et al., 2013; Srivastava and Baig, 2018; Mittelstadt and Ashwell, 2001; Nelson et al., 2003; Sch et al., 1995). Many studies have shown that glucocorticoids powerfully dampen chronic inflammation in dystrophic muscle, reducing muscle fiber injury, necrosis, and slowing disease progression, leading to improved strength and function (Kissel et al., 1993; Kissel et al., 1991; Wehling-Henricks et al., 2004). Intriguingly, a recent study has demonstrated that physiological endogenous GR signaling plays a protective role in muscular dystrophy. Deletion of GR in skeletal and cardiac muscle of mdx (a mouse model of DMD) mice worsens dystrophic phenotypes leading to reduced muscle strength and inflammation and exacerbate cardiomyopathy (Oliver et al., 2024).

Although effective, glucocorticoid treatment for DMD is accompanied by well-documented adverse effects such as osteoporosis, obesity, short stature, delayed puberty, and adrenal insufficiency (Quattrocelli et al., 2021; Fontaine Carb et al., 2024). Prolonged exposure to glucocorticoids can cause muscle wasting in individuals without muscular dystrophy or any other disease. High dosages of glucocorticoid augment the gene expression of various atrogenes, including muscle-specific E3 ubiquitin ligases, MuRF1 and MAFBx, potentially through the activation of FOXO family of transcription factors (Watson et al., 2012; Sandri et al., 2004). Intriguingly, elevated physiological glucocorticoid levels are also associated with muscle weakness across a range of conditions, including diabetes mellitus and metabolic acidosis (May et al., 1986; Hu et al., 2009). Efforts to develop novel glucocorticoid derivatives are ongoing, with the goal of reducing adverse effects and enhancing specific aspects of glucocorticoid activity, such as selective activation of the GR. Notable synthetic derivatives of glucocorticoid include CpdX, an anti-inflammatory GR agonist, and vamorolone (VBP-15), both of which demonstrate improved clinical efficacy in animal models and patients with DMD (Hua et al., 2019; Conklin et al., 2018; Heier et al., 2013). Furthermore, different treatment regimens and dosing are being tested to maximize the benefits and minimize side effects of glucocorticoid derivates in DMD patients (Quattrocelli et al., 2017; Fontaine Carb et al., 2024).

Glucocorticoids also confer clinical benefits in quantitative muscle strength testing among patients with Becker muscular dystrophy, limb-girdle muscular dystrophies, and other forms of muscular dystrophy (Quattrocelli et al., 2021). In addition, glucocorticoids are the first-line therapy for several autoimmune neuromuscular disorders, including inflammatory myopathies, myasthenia gravis, and chronic inflammatory demyelinating polyradiculoneuropathy, conditions that often necessitate long-term immunosuppression (Bacher et al., 2025).

**FIGURE 2:**
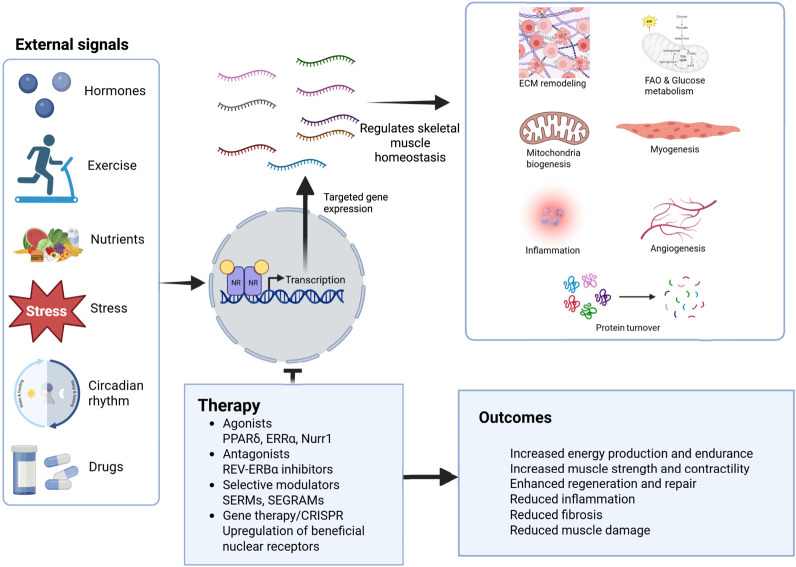
Nuclear receptor signaling regulates skeletal muscle homeostasis and therapeutic responses. External cues including hormones, exercise, nutrients, stress, circadian rhythm, and pharmacological agents modulate nuclear receptor (NR)-dependent transcriptional programs in skeletal muscle. Activation or inhibition of specific NRs regulates gene expressions involved in extracellular matrix remodeling, mitochondrial biogenesis, fatty acid oxidation and glucose metabolism, myogenesis, inflammation, angiogenesis, and protein turnover. Therapeutic targeting of NRs using agonists, antagonists, selective modulators, or gene therapy approaches may improve muscle energy metabolism, regeneration, strength, and reduce fibrosis and muscle damage in various muscle diseases, including muscular dystrophy. The image was created with BioRender.com.

In summary, glucocorticoids are potent therapeutic agents with broad clinical utility across neuromuscular diseases (Figures 1, 2). However, optimizing their use requires careful adaptation of treatment regimens to specific disease subtypes, as well as consideration of disease severity and stage of progression. Further research into the mechanisms underlying glucocorticoid action is essential to refine existing therapies and to expand the development and application of improved glucocorticoid derivatives for diverse neuromuscular conditions.

### 3.2 Mineralocorticoid receptor (MR)

The MR is a crucial ligand-activated nuclear transcription factor, primarily responding to the hormone aldosterone to regulate salt/water balance, blood pressure, and electrolyte levels in the kidneys. MR is also expressed in multiple tissues, including heart, vasculature, brain, skeletal muscle, and immune cells and regulate various physiological processes, like inflammation, fibrosis, and neuromuscular function. Upon ligand binding, MR translocates into the nucleus and binds to enhancer or promoter regions, controlling the expression of its target genes (Lother et al., 2015). MR has structural and functional similarities to GR and therefore it has equal affinity for mineralocorticoids and glucocorticoids. Both receptors can be co-expressed within the same cell, and it is believed that the balance in GR and MR expression is a key for maintaining homeostasis and adaptation to stress signals (Fuller et al., 2025). MR selectivity for aldosterone is largely mediated by type 2 11β-hydroxysteroid dehydrogenase (11β-HSD2), which inactivates glucocorticoids and prevents their binding to the MR (Lother et al., 2015).

While the classical role of MR is to regulate blood pressure and salt-water homeostasis, chronic overactivation of MR by its natural ligand aldosterone is a key driver of cardiovascular diseases. MR antagonists reduce mortality and morbidity in patients with heart failure by ameliorating multiple pathological processes, including oxidative stress, apoptosis, inflammation, fibrosis, cardiomyocyte hypertrophy, electrical remodeling, and endothelial dysfunction (Lother et al., 2015; Mizuno et al., 2001; Lother and Hein, 2016). Preclinical studies have shown that early treatment with the aldosterone antagonist spironolactone, in combination with the angiotensin-converting enzyme inhibitor lisinopril, reduces tissue injury and fibrotic accumulation and improves functional outcomes in both cardiac and skeletal muscles in mouse models of DMD (Rafael-Fortney et al., 2011; Lowe et al., 2016). Moreover, clinical studies indicate that early initiation of MR antagonist therapy delays the onset and progression of cardiac dysfunction in patients with DMD (Duboc et al., 2005; Raman et al., 2017; Raman et al., 2019) (Table 1).

Most aldosterone is synthesized in the adrenal cortex. However, aldosterone and glucocorticoids can be synthesized locally in extra-adrenal tissues, including skeletal muscle, especially in pathological states (Taves et al., 2011; Chadwick et al., 2015). Increased expression of aldosterone synthase (gene name: CYP11B2), the key enzyme in aldosterone synthesis has been observed in the failing heart ventricles in humans which may be the reason for the observed local upregulation of aldosterone during heart failure (Mizuno et al., 2001). To understand the mechanisms behind the efficacy of MR antagonists in dystrophic muscles, Chadwick et al. investigated the production of aldosterone by different cell types present in dystrophic muscle microenvironment and reported that infiltrating leukocytes are one of the important sources for the production of aldosterone (Chadwick et al., 2016). Cell culture studies showed that genes encoding various enzymes in the pathway for aldosterone synthesis are expressed in muscle-derived leukocytes. Furthermore, the levels of 11β-HSD2 enzyme, which inactivates glucocorticoids to increase MR selectivity for aldosterone, are also elevated in dystrophic muscle tissues suggesting that local production of aldosterone contributes to the pathology of muscular dystrophy (Chadwick et al., 2016). Intriguingly, some levels of MR signaling in myeloid cells are essential for regeneration of normal skeletal muscle in response to acute injury and to prevent accumulation of excessive fibrosis in diaphragm of mdx mice (Howard et al., 2022a).

To further decipher the mechanisms through which MR signaling contributes to dystrophic phenotype, Howard et al. investigated the effects of MR antagonist spironolactone and glucocorticoid prednisolone in regulation of inflammation in dystrophic muscle of *mdx* mice (Howard et al., 2022b). The study showed that spironolactone alleviates inflammation without altering leukocyte distribution within skeletal muscles. In contrast, prednisolone suppresses quadriceps cytokines but increases diaphragm cytokines and pathology (Howard et al., 2022b). While more investigations are needed to understand the contribution of MR signaling in dystrophic muscle, temporal or combinatorial modulation of GR and MR signaling may represent a strategy to maximize therapeutic benefit while limiting adverse effects.

### 3.3 Peroxisome proliferator-activated receptor (PPARs)

PPARs are a family of ligand-activated nuclear hormone receptors that orchestrate gene expression programs governing metabolism, inflammation, and energy homeostasis (Grygiel-Gorniak, 2014). The PPAR family comprises three major isoforms with distinct tissue distribution and functional roles. PPARα is highly expressed in liver, heart, and skeletal muscle, where it promotes fatty acid oxidation during fasting. PPARγ is predominantly expressed in adipose tissue and regulates adipogenesis, lipid storage, and insulin sensitivity. In contrast, PPARδ (PPARβ/δ) is ubiquitously expressed and drives fatty acid utilization, energy expenditure, and oxidative/endurance metabolic programs (Manickam et al., 2020; Forman et al., 1996). PPARs are activated by lipid-derived ligands, including fatty acids and their metabolites. Upon activation, they heterodimerize with the retinoid X receptor (RXR) and bind to PPAR response elements (PPREs) within target gene promoters to regulate transcription. Ligand-bound PPARs recruit transcriptional coactivators such as PPARγ coactivator-1α (PGC-1α) and PGC-1β, thereby amplifying transcriptional output and coordinating mitochondrial biogenesis and oxidative metabolic gene networks (Grygiel-Gorniak, 2014; Forman et al., 1996).

Although the role of PPARs in muscular disorders has been less extensively characterized than in metabolic diseases, a growing body of preclinical evidence highlights their therapeutic potential in DMD. Pharmacological activation of PPARδ using small-molecule agonists has been shown to restore fatty acid oxidation capacity in mdx myoblasts by upregulating genes involved in lipid utilization, establishing PPARδ as a key metabolic target in dystrophic muscle (Bell et al., 2019). Importantly, the dystrophin homolog utrophin A can functionally compensate for dystrophin loss. Activation of PPARβ/δ, for example, using GW501516, induces a slow oxidative fiber-type program and increases utrophin A expression, thereby improving sarcolemmal stability and reducing muscle damage in mdx mice (Miura et al., 2009).

Further supporting the importance of metabolic remodeling, combined interventions have demonstrated synergistic effects. Co-administration of the AMPK activator AICAR and the PPARδ agonist GW1516, together with exercise training, significantly improved muscle performance, enhanced oxidative metabolism markers, and reduced serum creatine kinase levels compared to single interventions (Bueno Junior et al., 2012). These findings underscore the benefit of integrating metabolic and functional stimuli to improve dystrophic pathology.

Additional pharmacological approaches have reinforced the relevance of PPAR-linked signaling networks. Another study showed that MA-0204, a selective PPARδ modulator, improves the dystrophic phenotype in mdx mice by enhancing mitochondrial fatty acid oxidation and activating oxidative metabolic gene programs, thereby restoring bioenergetic capacity in dystrophic muscle (Lagu et al., 2018). PSB-KD107, a synthetic agonist of the resolvin D2 receptor GPR18, enhances myogenic differentiation in patient-derived iPSC myoblasts and reduces inflammation while promoting regeneration in mdx mice. Mechanistically, these effects are mediated through coordinated activation of PPARs, AMPK, mTOR, and sphingolipid signaling pathways (Dort et al., 2023). Together, these studies highlight the central role of PPAR-driven metabolic reprogramming in improving dystrophic muscle function.

Genetic and metabolic studies further support the role of the PPAR-PGC-1α axis in muscular dystrophy. Transgenic overexpression of PGC-1α in skeletal muscle of mdx mice improves muscle histopathology, enhances exercise capacity, and reduces serum creatine kinase levels. PGC-1α interacts with the GA-binding protein (GABP) complex to promote transcription of neuromuscular junction gene programs (Handschin et al., 2007). Consistent with these findings, metformin, an AMPK activator widely used in type 2 diabetes, has been shown to increase PGC-1α, PPARδ, and utrophin A expression in cultured muscle cells and dystrophic muscle in mdx mice (Ljubicic and Jasmin, 2015).

Similar mechanisms operate in other muscular dystrophies. Loss-of-function mutations in calpain-3, which cause limb-girdle muscular dystrophy type 2A, are associated with mitochondrial dysfunction. Treatment of calpain-3-deficient mice with the PPARδ agonist GW501516 enhances mitochondrial biogenesis, increases expression of myogenic regulators such as MyoD and Pax7, and reduces muscle fatigability and serum creatine kinase levels. Restoration of mitochondrial metabolism with pyruvate further improves sarcolemmal repair in dystrophic muscle (Jahnke et al., 2020).

Despite the beneficial effects of PPAR activation, its role in muscle pathology is context-dependent. Intramuscular adipose tissue negatively regulates myogenesis in skeletal muscle disorders such as DMD. Suppression of PPARγ-mediated adipogenic differentiation enhances myotube formation, whereas mature adipocytes impair myogenesis, suggesting that adipocyte maturation contributes to muscle degeneration (Takegahara et al., 2014).

Building on this strong preclinical rationale, PPARδ agonists have advanced into clinical development. ASP0367 (bocidelpar sulfate), an orally bioavailable and selective PPARδ modulator, was designed to target mitochondrial dysfunction and metabolic impairment in DMD and primary mitochondrial myopathies. Early-phase studies demonstrated favorable safety, pharmacokinetic properties, and dose-dependent induction of PPARδ target genes in healthy participants (Ito et al., 2022). However, subsequent clinical evaluation revealed limited therapeutic benefit. The phase 1b study in boys with DMD was constrained by small sample size and early termination, limiting efficacy interpretation. More notably, the phase 2 MOUNTAINSIDE trial in primary mitochondrial myopathy failed to meet its primary efficacy endpoint, showing no meaningful improvement in functional outcomes. Consequently, despite acceptable tolerability and evidence of pharmacodynamic target engagement, clinical development of ASP0367 was discontinued (Table 1).

Collectively, these findings position the PPAR-PGC-1α axis as a central regulator of metabolic remodeling of dystrophic muscle and a promising, albeit complex, therapeutic target. The discrepancy between robust preclinical efficacy and limited clinical success highlights the need for improved translational strategies and combinatorial approaches in targeting metabolic pathways in muscular dystrophies.

### 3.4 REV-ERB family

The REV-ERB family comprises two closely related heme-binding nuclear receptors, REV-ERBα (NR1D1) and REV-ERBβ (NR1D2), which are key regulators of circadian rhythms, metabolism, and inflammatory responses (Yin et al., 2007; Kojetin and Burris, 2014). Unlike classical nuclear receptors that function as transcriptional activators, REV-ERBs act as ligand-dependent transcriptional repressors. They lack a conventional activation function (AF-2) domain and instead repress gene expression by recruiting corepressor complexes, including NCoR and HDAC3 (Kojetin and Burris, 2014). As integral components of the molecular circadian clock, REV-ERBα and REV-ERBβ suppress core clock genes such as BMAL1, thereby helping to sustain ∼24-h oscillatory patterns of gene expression (Yin et al., 2007; Cho et al., 2012; Bugge et al., 2012).

Brain and Muscle ARNT-Like 1 (BMAL1), a central circadian regulator, promotes myogenic differentiation through direct transcriptional activation of canonical Wnt signaling components, linking the clock to muscle development. Genetic ablation of BMAL1 in mice reduces total muscle mass, and Bmal1-deficient primary myoblasts show markedly impaired differentiation with blunted expression of key myogenic regulatory factors and Wnt pathway components (Chatterjee et al., 2013). In addition, BMAL1 is required for maintenance of the satellite cell population and for skeletal muscle regeneration after injury (Chatterjee et al., 2015). REV-ERB represses Bmal1 *via* REV-ERB response element (RORE)-dependent promoter binding and also inhibits myoblast differentiation through RORE-independent suppression of myogenic and cell cycle pathways. Partial loss of Rev-Erbα or pharmacologic disruption of its repressive activity accelerates skeletal muscle repair after injury (Welch et al., 2017a). Consistent with its negative role in myogenesis, antagonism of REV-ERB with SR8278 in mdx mice improves muscle function, reduces fibrosis, and enhances mitochondrial biogenesis and an oxidative fiber phenotype (Welch et al., 2017b). Similarly, genetic ablation of Rev-Erbα in mdx mice improves dystrophic pathology by promoting myogenesis and reducing inflammatory responses (Xiong et al., 2021). Collectively, these studies suggest that inhibition of REV-ERB activity may represent a therapeutic strategy for muscular dystrophy.

The sarcoplasmic reticulum (SR) is essential for calcium homeostasis, and its dysfunction is a hallmark of myopathy. Notably, REV-ERBα regulates skeletal muscle calcium handling by enhancing sarco/endoplasmic reticulum calcium ATPase (SERCA)-dependent SR calcium uptake through repression of the SERCA inhibitor myoregulin. Loss of REV-ERBα impairs calcium handling, whereas its activation restores SR function and improves muscle structure and performance in a mouse model of DMD (Boulinguiez et al., 2022). These findings further underscore REV-ERBα as a promising therapeutic target for myopathies.

### 3.5 Vitamin D receptors (VDRs)

VDRs are ligand-activated nuclear hormone receptors that mediate the biological effects of vitamin D in the body. The active form of vitamin D, 1,25-dihydroxyvitamin D_3_ (calcitriol), binds to VDR to regulate gene expression (Toell et al., 2000). Once activated, VDR forms a heterodimer with the retinoid X receptor (RXR) and binds to specific DNA sequences called vitamin D response elements (VDREs) in target genes. This complex functions mainly as a transcriptional regulator, controlling genes involved in calcium and phosphate homeostasis, immune regulation, and cell growth (Toell et al., 2000).

Beyond its classical role in bone and mineral metabolism, VDR is expressed in many tissues, including skeletal muscle, where it influences muscle cell proliferation, differentiation, and function by supporting the maturation and differentiation of myogenic cells (Polly and Tan, 2014; Srikuea et al., 2012; Irazoqui et al., 2014). Dysregulation of VDR signaling has been linked to muscle weakness, sarcopenia, and impaired muscle regeneration following injury (Srikuea et al., 2012; Irazoqui et al., 2014; Endo et al., 2003). For example, Vitamin D deficiency results in a reduction in skeletal muscle mass and function and its supplementation enhances muscle function and myofiber size in the elderly (Dhanwal et al., 2013; Ceglia et al., 2013). Anabolic role of VDR in skeletal muscle is supported by the findings that overexpression of VDR in rodent muscle stimulates muscle hypertrophy through enhancing protein synthesis, ribosomal expansion, and extracellular matrix remodeling (Bass et al., 2020). Intriguingly, Vitamin D-independent functions of the VDR have also been described in skeletal muscle, where reduced VDR protein expression is associated with aging and multiple disease states (Bischoff-Ferrari et al., 2004).

Transcriptomic and microRNA-based analyses have identified dysregulation of vitamin D receptor (VDR)-associated signaling pathways in muscular dystrophies, including limb-girdle muscular dystrophy, suggesting altered vitamin D responsiveness at the molecular level (Aguennouz et al., 2016). In fact, vitamin D supplementation has been investigated as a potential adjunct strategy to improve skeletal muscle function in DMD, given its established role in muscle physiology and the high prevalence of vitamin D deficiency in patients. However, preclinical evidence remains inconsistent. In a recent study, short-term (4-week) vitamin D supplementation across a range of doses failed to improve muscle contractile properties, fatigue resistance, or histopathological indices in *mdx* mice, despite a modest increase in muscle mass at higher doses (Debruin et al., 2019). These findings are in line with other reports demonstrating limited efficacy of vitamin D as a standalone intervention in dystrophic muscle, including studies showing minimal effects of vitamin D deficiency or supplementation on muscle outcomes in *mdx* models (Yoon et al., 2018) and broader analyses indicating no consistent improvement in muscle strength or performance with supplementation (Bislev et al., 2021). Collectively, these data indicate that while vitamin D may support overall musculoskeletal health, its direct impact on dystrophic muscle pathology is modest, supporting a primarily adjunctive rather than disease-modifying role in DMD; consistent with clinical evidence suggesting supplementation mainly corrects deficiency without robust effects on disease progression (Guo and Anthony, 2023).

Dysferlinopathy is a heterogeneous group of autosomal recessive muscular dystrophies caused by mutations in the *DYSF* gene (Fanin and Angelini, 2016). Dysferlin is predominantly expressed in skeletal muscle and peripheral blood monocytes (PBMs), and affected patients typically exhibit markedly reduced or absent protein levels. One study demonstrated that vitamin D3 treatment increases dysferlin expression in cultured cells as well as in PBMs of patients with dysferlinopathy, suggesting that combining vitamin D3 supplementation with complementary molecular strategies may enhance dysferlin expression in affected individuals (De Luna et al., 2012).

Myotonic dystrophy is a multisystemic, autosomal dominant disorder caused by CTG (DM1) or CCTG (DM2) repeat expansions, characterized by progressive muscle weakness, myotonia, and widespread endocrine and metabolic dysfunction. Vitamin D deficiency is common in myotonic dystrophy and correlates with disease severity. It is linked to reduced IGF-1 signaling, weaker muscle strength, and secondary hyperparathyroidism, suggesting a role in muscle dysfunction (Terracciano et al., 2013). However, since DM is primarily driven by RNA toxicity and splice defects, vitamin D is likely to act as a modifier of endocrine and metabolic pathways rather than a direct cause of muscle degeneration. Supplementation may improve systemic and skeletal outcomes, but its effect on intrinsic muscle pathology is limited (Passeri et al., 2013).

Collectively, these findings highlight VDR signaling as an important endocrine-metabolic regulator of skeletal muscle homeostasis with context-dependent effects across neuromuscular diseases. However, current evidence indicates that VDR-targeted interventions are most effective as adjunctive strategies, complementing disease-specific therapies rather than directly modifying primary dystrophic pathology.

### 3.6 Thyroid hormone receptors (TRs)

TRs are ligand-activated nuclear receptors that mediate the biological effects of thyroid hormones, primarily triiodothyronine (T3) and, to a lesser extent, thyroxine (T4). The genes THRA and THRB encode multiple receptor isoforms (e.g., TRα and TRβ), which function as transcription factors by binding to thyroid hormone response elements (TREs) in target genes. In the absence of ligand, TRs typically repress gene transcription, whereas ligand binding induces conformational changes that promote coactivator recruitment and transcriptional activation (Cheng et al., 2010).

TR signaling plays a central role in skeletal muscle homeostasis by regulating contractile function, mitochondrial metabolism, and regenerative capacity (Bloise et al., 2018). In hypothyroid states, reduced intramuscular T3 impairs energy metabolism and excitation-contraction coupling, leading to muscle weakness, cramps, and elevated creatine kinase (CK) levels that are largely reversible with hormone replacement (Rodolico et al., 1998). Conversely, excessive TH signaling can exacerbate neuromuscular dysfunction, including increased fatigability and autoimmune activity in disorders such as myasthenia gravis, highlighting the importance of tightly regulated TH homeostasis (Rodolico et al., 1998).

In muscular dystrophies, TR signaling functions primarily as a context-dependent modifier of disease progression. THs critically regulate satellite cell activation, myogenesis, and muscle regeneration, and both deficiency and excess can impair regenerative processes and influence disease outcomes in conditions such as DMD (Bloise et al., 2018; Milanesi et al., 2017; Milanesi et al., 2016). The complexity of TR signaling in neuromuscular disease is further illustrated by rare genetic disorders such as resistance to thyroid hormone β (RTHβ), caused by mutations in the THRB gene that result in impaired tissue responsiveness despite elevated circulating thyroid hormone levels and nonsuppressed TSH (Bayraktaroglu et al., 2009). In contrast, myotonic dystrophy type 1 (DM1) arises from CTG repeat expansion in the DMPK gene, leading to RNA-mediated splice dysregulation and multisystem endocrine dysfunction. Takeda et al. identified P453A and C36Y THRB mutations in a family with coexisting DM1, demonstrating that these are independent genetic conditions with overlapping endocrine phenotypes, complicating the interpretation of thyroid function (Takeda et al., 2019). These findings emphasize the importance of genetic evaluation in atypical thyroid presentations, particularly in multisystem disorders, to avoid misdiagnosis and inappropriate therapeutic interventions.

TR signaling is also implicated in other myopathies, including selenoprotein-related disorders and rhabdomyolysis, largely through its regulation of mitochondrial function, oxidative metabolism, and muscle repair pathways (Bloise et al., 2018; Sat et al., 2007). Collectively, these observations highlight TR signaling as a critical regulator of skeletal muscle integrity, with dysregulation contributing to, or exacerbating, neuromuscular disease.

### 3.7 Estrogen-related receptors (ERRs)

ERRs are orphan nuclear receptors that function as constitutively active transcription factors. Despite their nomenclature, they do not bind estrogen but instead regulate gene networks controlling mitochondrial biogenesis, oxidative metabolism, and energy homeostasis, often in cooperation with coactivators such as PGC-1α/β (Frigo et al., 2021; Kumar and Narkar, 2023). ERRs have emerged as central regulators of skeletal muscle homeostasis, with ERRα and ERRγ being the predominant isoforms in muscle. These receptors integrate environmental and metabolic cues, including exercise, fasting, hypoxia, and nutrient availability to coordinate transcriptional programs governing muscle function. In contrast, pathological conditions such as diabetes and muscular dystrophy are associated with reduced ERR expression, which may contribute to impaired metabolic adaptation and disease progression (Frigo et al., 2021; Mangelsdorf et al., 1995; Kumar and Narkar, 2023).

Mitochondrial dysfunction and reduced oxidative capacity are key features of dystrophic muscle (Shin et al., 2013; Vila et al., 2017). Consistently, ERRγ and its downstream metabolic and angiogenic targets are markedly downregulated in mdx muscle, accompanied by reduced oxidative myofibers, vascularization, and exercise capacity. Restoration of ERRγ signaling *via* muscle-specific overexpression rescues these defects, enhancing mitochondrial function, angiogenesis, and exercise tolerance while reducing muscle damage and fatigue (Matsakas et al., 2013). Similarly, Hardee et al. demonstrated that ERRα-dependent transcriptional programs are impaired in dystrophic muscle, particularly those involved in oxidative phosphorylation and mitochondrial function, linking defective metabolic remodeling to disease pathology (Hardee et al., 2021).

Recent work further highlights the role of ERRα in muscle regeneration. ERRα is induced during myoblast differentiation and in regenerating muscle, where it promotes myogenic progression, angiogenesis, and mitochondrial biogenesis. Notably, ERRα expression and its downstream programs are suppressed in dystrophic muscle and muscle stem cells, contributing to impaired regeneration. Muscle-specific activation of ERRα restores angio-metabolic signaling, enhances regeneration, reduces muscle damage, and improves functional outcomes in mdx mice (Nguyen et al., 2025).

Pharmacological targeting of ERRs is emerging as a promising therapeutic strategy. The ERR pan-agonist SLU-PP-915 is orally bioavailable and exhibits exercise-mimetic activity, enhancing mitochondrial gene expression and oxidative metabolism *in vivo* (Billon et al., 2026). Although not yet tested in muscular dystrophy models, such compounds have the potential to reprogram muscle metabolism and attenuate disease progression.

Beyond DMD, ERR signaling is also implicated in other types of muscular dystrophies. In facioscapulohumeral muscular dystrophy (FSHD), which is driven by aberrant DUX4 expression, impaired myogenesis is associated with suppression of the PGC-1α-ERRα axis. Restoration of this pathway using ERRα agonists improves myogenic differentiation and rescues muscle defects (Banerji et al., 2019). Collectively, these findings position ERRs as key regulators of metabolic, angiogenic, and regenerative programs, and highlight their therapeutic potential in muscular dystrophies.

### 3.8 Chicken ovalbumin upstream promoter-transcription factor II (COUP-TFII)

COUP-TFII, also known as NR2F2, is an orphan NR which was originally identified by its ability to bind regulatory regions upstream the ovalbumin gene (Ashraf et al., 2019). COUP-TFII regulates gene expression by binding DNA at specific response elements and recruiting co-regulators, typically acting as a transcriptional repressor, though it can also activate genes depending on context. It plays critical roles in embryonic development, including angiogenesis, heart formation, organogenesis, and metabolic regulation, and is also involved in cell fate decisions and differentiation (Ashraf et al., 2019; Polvani et al., 2019).

COUP-TFII is required for proper development of skeletal musculature in the limbs (Lee et al., 2004). Its expression is high in undifferentiated progenitors and declines during differentiation, a pattern necessary for efficient myoblast fusion. It has been reported that COUP-TFII inhibits myoblast fusion by repressing gene expression of Nephronectin, Integrin Subunit Beta 1, and Caveolin-3 and inhibiting the activation of focal adhesion kinase, all of which are crucial for the fusion process (Lee et al., 2017). It also suppresses satellite cell proliferation by inhibiting multiple myogenic regulatory factors (Xie et al., 2016; Aguiari et al., 2021). Repression of COUP-TFII preserves satellite cell function and alleviates muscle weakness in mdx mice, whereas ectopic expression in satellite cells induces progressive myopathy in control mice and worsens pathology in mdx mice (Xie et al., 2016). Together, these findings implicate COUP-TFII in the pathogenesis of muscular dystrophy and support its inhibition as a potential therapeutic strategy for DMD.

### 3.9 Other nuclear receptors

Several additional nuclear receptors regulate skeletal muscle metabolism and exercise capacity. Nuclear receptor 4A2 (NR4A2; Nurr1), a member of the NR4A orphan receptor family (with NR4A1/Nur77 and NR4A3/NOR1), functions as a ligand-independent, immediate-early transcription factor induced by stress, growth factors, and exercise. Muscle-specific overexpression of Nurr1 enhances physical performance and improves systemic metabolism by increasing glucose uptake and glycogen storage, thereby preventing hyperglycemia and hepatic steatosis. Pharmacological activation of Nurr1 similarly increases energy expenditure, improves glucose tolerance, and promotes a lean phenotype, mimicking exercise (Amoasii et al., 2019).

Retinoic acid receptor-related orphan receptor-α (RORα; NR1F1) is another orphan nuclear receptor that acts as a ligand-regulated transcription factor, binding ROR response elements (ROREs) to control gene programs involved in circadian rhythm, lipid metabolism, inflammation, and differentiation (Coo et al., 2015). RORα supports oxidative metabolism and mitochondrial function, contributing to the maintenance of oxidative muscle fibers (Kim et al., 2024), and also regulates myogenic differentiation and muscle repair through crosstalk with circadian regulators such as BMAL1 and REV-ERB. Although the roles of Nurr1 and RORα in muscular dystrophies are not yet fully defined, they likely modulate muscle metabolism and inflammation, highlighting their potential as therapeutic targets in muscular disorders. Together, these findings highlight the diverse and context-dependent roles of NRs in skeletal muscle biology and underscore their potential as therapeutic targets across a broad spectrum of neuromuscular diseases.

## 4 Conclusions and future perspectives

NRs have emerged as central integrators of endocrine, metabolic, and stress signals that coordinate skeletal muscle homeostasis and disease progression. Across diverse receptor classes, emerging evidence demonstrates that NRs regulate key processes including mitochondrial biogenesis, oxidative metabolism, inflammation, satellite cell function, and muscle regeneration. Disruption of NR signaling is a common feature of muscular dystrophies and other myopathies, where impaired NR activity contributes to metabolic dysfunction, chronic inflammation, fibrosis, and defective regeneration. Importantly, both preclinical and clinical studies indicate that pharmacological or genetic modulation of specific NRs can improve muscle structure and function, underscoring their strong translational potential (Figure 2).

Despite these advances, several key gaps remain in our understanding of NR signaling in muscle physiology and disease. The context-dependent and pleiotropic actions of NRs across different cell populations within skeletal muscle are still incompletely defined. In addition, cross-regulatory interactions among NRs highlight that skeletal muscle signaling is governed by integrated transcriptional networks rather than isolated pathways. Interplay between GR–MR, TR–VDR, and the PPAR-ERR-PGC-1α axis coordinates inflammatory, metabolic, and regenerative processes, and their dysregulation contributes to muscle pathology. These observations have important therapeutic implications, suggesting that combinatorial targeting of NR networks or shared co-regulators (e.g., PGC-1α modulators or selective dual NR ligands) may offer greater efficacy than single-receptor approaches. Future work should therefore prioritize network-based or pathway-integrated strategies to restore coordinated NR signaling in neuromuscular diseases.

Another key limitation is that despite strong preclinical evidence supporting multiple nuclear receptor pathways, clinical translation remains largely confined to GR and MR-targeting therapies. In contrast, other NRs, including PPARs, ERRs, TRs, and VDR have yet to demonstrate clinical efficacy in controlled trials, highlighting a significant gap between mechanistic insights and therapeutic application. It is also notable that mouse models, particularly the mdx mouse, have provided critical mechanistic insights but differ substantially from human DMD in disease severity, regenerative capacity, fibrosis, and lifespan. These species-specific differences along with divergences in inflammatory, transcriptional, and NR signaling networks limit direct translation of preclinical findings to patients. Therefore, integrating human-relevant systems and cross-species validation will be essential to improve the translational impact of therapeutic strategies.

Future research should also focus on cell type-specific and stage-specific mapping of NR signaling in both healthy and diseased muscle. Integration of multi-omics approaches to define NR-regulated gene networks, along with the development of selective, tissue-targeted NR modulators with improved safety profiles, will be essential. Ultimately, a more comprehensive understanding of NR signaling will facilitate the translation of these pathways into effective therapies for muscular dystrophies and related neuromuscular conditions.

## 5 Methods

### 5.1 Literature search and selection Criteria

A comprehensive literature search was performed using PubMed, Scopus, and Google Scholar to identify relevant studies published between January 1990 and May 2026. Search terms included combinations of keywords and Medical Subject Headings (MeSH), such as “muscular dystrophy,” “Duchenne muscular dystrophy,” “myotonic dystrophy,” “nuclear receptor,” “PPAR,” “glucocorticoid receptor,” “Mineralocorticoid receptor,” “Peroxisome proliferator-activated receptor,” “Vitamin D receptor,” “thyroid hormone receptor,” “REV-ERB,” “COUP-TFII”, “estrogen-related receptor,” and related terms, combined using Boolean operators (AND/OR). Only peer-reviewed articles published in English were considered. Eligible studies included original research articles and reviews investigating the role of nuclear receptors in muscular dystrophies, including mechanistic, preclinical, and clinical studies. Studies focusing exclusively on nuclear receptor function in normal muscle physiology without relevance to disease contexts were excluded. Titles and abstracts were screened for relevance, followed by full-text evaluation of selected articles. Reference lists of key publications were also manually screened to identify additional relevant studies. The literature included was qualitatively synthesized using a thematic analysis approach, in which studies were grouped based on specific nuclear receptor and its functional roles in disease pathogenesis, inflammation, metabolism, and muscle regeneration. Emerging patterns and cross-regulatory mechanisms were identified and integrated to provide a comprehensive overview of nuclear receptor signaling in muscular dystrophy.
